# A comparison of patient recall of smoking cessation advice with advice recorded in electronic medical records

**DOI:** 10.1186/1471-2458-11-291

**Published:** 2011-05-10

**Authors:** Lisa Szatkowski, Ann McNeill, Sarah Lewis, Tim Coleman

**Affiliations:** 1 Division of Primary Care, University of Nottingham, Queen's Medical Centre, Nottingham, NG7 2UH, UK; 2 Division of Epidemiology and Public Health, University of Nottingham, Clinical Sciences Building, Nottingham City Hospital, Nottingham, NG5 1PB, UK

**Keywords:** smoking, family practice, medical records, computer systems

## Abstract

**Background:**

Brief cessation advice delivered to smokers during routine primary care consultations increases smoking cessation rates. However, in previous studies investigating recall of smoking cessation advice, smokers have reported more advice being received than is actually documented in their medical records. Recording of smoking cessation advice in UK primary care medical records has increased since the introduction of the Quality and Outcomes Framework (QOF) in 2004, and so we compare recall and recording of cessation advice since this time to assess whether or not agreement between these two data sources has improved.

**Methods:**

For each year from 2000 to 2009, the proportion of patients in The Health Improvement Network Database (THIN) with a recording of cessation advice in their notes in the last 12 months was calculated. In 2004, 2005 and 2008, these figures were compared to rates of patients recalling having received cessation advice in the last 12 months in the Primary Care Trust (PCT) Patient Surveys, with adjustment for age, sex and regional differences between the populations.

**Results:**

In 2004 there was good agreement between the proportion of THIN patients who had cessation advice recorded in their medical records and the proportion recalling advice in the Patient Survey. However, in both 2005 and 2008, more patients had cessation advice recorded in their medical records than recalled receiving advice.

**Conclusions:**

Since the introduction of the QOF, the rate of recording of cessation advice in primary care medical records has exceeded that of patient recall. Whilst both data sources have limitations, our study suggests that, in recent years, the proportion of smokers being advised to quit by primary care health professionals may not have improved as much as the improved recording rates imply.

## Background

The delivery of brief smoking cessation advice during routine general practice consultations, a simple intervention usually lasting no more than one or two minutes, has been shown to be effective in increasing cessation rates[1], and is one of the most cost-effective means to reduce the burden of smoking[2]. However, in the past, large discrepancies have been reported between the proportion of patients who recall having received cessation advice and the proportion of patients with advice documented in their medical records; in one study, cessation advice was recorded in the notes of just 30.9% of patients who reported having received advice[3].

The introduction of the Quality and Outcomes Framework (QOF) in April 2004 provided, for the first time, a financial incentive for GPs to document in smokers' medical records that they have been offered cessation advice, worth approximately £4,500 to each general practice per year[4]. Specifically, since 2004 GPs have been rewarded for recording having offered cessation advice to smokers with specified chronic health conditions (asthma, chronic obstructive pulmonary disease, chronic kidney disease, coronary heart disease, diabetes mellitus, hypertension, schizophrenia, bipolar affective disorder and other psychoses and stroke or transient ischemic attack) at least every 15 months[4]. A tripling of rates of advice recorded in medical records occurred in the year following the introduction of the QOF, with no concomitant increase in prescribing of nicotine replacement therapy or bupropion for which there is no QOF incentive[5]. One explanation for this is that the QOF simply increased GPs' propensity to document cessation advice offered, rather than actually increasing their rate of advice-giving[5]. To investigate whether this might have occurred we compared advice recorded in a large dataset of primary care records with that recalled in a survey of National Health Service (NHS) patients in England in the years since the introduction of the QOF.

## Methods

The Health Improvement Network (THIN) is a database containing the electronic primary care medical records of almost seven million patients from 446 general practices throughout the UK, and is broadly representative of the UK population in terms of patient demographic characteristics[6]. For each year from 2000 to 2009, all patients from the THIN dataset who were over the age of 16 and registered with an English practice on an index date of 1st July in that year were identified. Each patient's year of birth, sex and the Strategic Health Authority (SHA) within which their GP surgery was located were identified. Patients' medical records were searched for Read codes[7] documenting the delivery of smoking cessation advice to that patient, and, for each year, the proportion of patients with a recording of cessation advice in the 12 months prior to the index date was calculated.

The Primary Care Trust (PCT) Patient Surveys monitor patients' experiences of NHS services[8]. In 2004, 2005 and 2008, a simple random sample of patients was selected from each PCT in England, and a postal questionnaire administered asking whether the respondent had 'definitely' or 'to some extent' received cessation advice from a health professional at their GP surgery within the last 12 months. Completed questionnaires were received from 122,113 patients in 2004, 116,939 in 2005 and 69,470 in 2008 (response rates of 47.4%, 45.4% and 38.3% respectively).

Previous work using the Patient Survey has shown that the provision of smoking cessation advice by primary health care professionals varies with patient sex and age[9]. Consequently, as Patient Survey respondents and patients in the THIN dataset have different demographic characteristics, directly comparing 'raw' data on smoking cessation advice received by patients in each source is not appropriate. Therefore, we used the following standardisation procedure to enable comparison of data from THIN and the Patient Surveys. For 2004, 2005 and 2008, age group, sex and SHA-specific rates of patients reporting having received smoking cessation advice within the last 12 months at least 'to some extent' were calculated from Patient Survey responses. These rates were applied to strata of the THIN population (similarly defined by age group, sex and SHA) at the corresponding index date using indirect standardisation[10], producing estimates for annual rates of recalled cessation advice that might be expected from THIN patients, based on Patient Survey responses (referred to as 'predicted recall rates'). Predicted recall rates were then compared graphically with the actual cessation advice rates documented in THIN patients' medical records.

This study was approved by the Leicestershire and Rutland Research Ethics Committee.

All analyses were completed using STATA version 11.0 (STATA Corp, College Station, TX).

## Results

**Figure 1:**
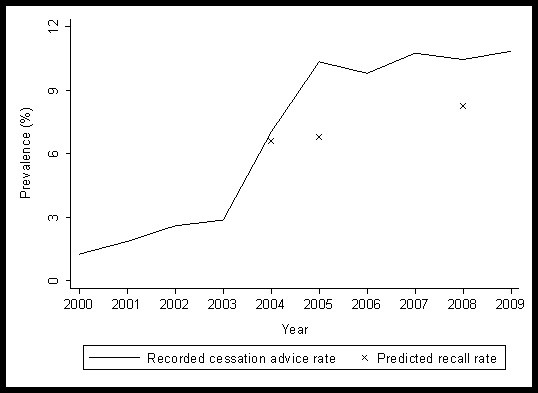
**The proportion of THIN patients aged 16+ with recorded cessation advice and predicted recall rates (given the extremely large sizes of the THIN dataset and Patient Surveys, the 95% confidence intervals around data points on the figure are within +/- 0.4% of point estimates and so are not shown)**.

The number of patients aged 16+ registered with a THIN practice in England increased from 1.8 million in July 2000 to 2.0 million in July 2009. At each index date, 49% of patients were male, with a mean age of 47 years (interquartile range 32-61). Figure 1 shows, for years studied, the proportions of patients within THIN who had smoking cessation advice documented in their medical records in the previous 12 months and, for Patient Survey years, predicted recall rates (estimates for annual rates of recalled cessation advice within THIN patients, based on the results of the Patient Survey).

The proportion of THIN patients with cessation advice documented in their medical records increased considerably over the study period, from 1.2% of patients in 2000 to 10.9% in 2009, with the majority of this increase occurring between 2003 and 2005. However, although similar in 2004, the predicted recall rate was subsequently lower and increased less over the survey period (6.6% of patients in 2004; 8.3% in 2008). In 2004 there was good agreement between recording of cessation advice in THIN and predicted recall rates, but in both 2005 and 2008 agreement between recorded and predicted recall of cessation advice was much less strong with recall rates being much lower.

## Discussion and conclusions

This study is, to our knowledge, the first to compare, at a population-level, smoking cessation advice recorded in medical records with patients' recall of such advice as reported in large surveys; in 2004 there was close agreement between both data sources but this decreased substantially in 2005 and 2008.

Some of the longitudinal changes in the proportions of patients recalling cessation advice, or having this documented in their medical records, may be due to changes in population smoking prevalence. Ideally, this study would have assessed recorded and recalled advice within smokers only, but the ability to confidently identify smokers in THIN was poor in the early years of this analysis[11] and it was impossible to identify respondents who were smokers from the 2008 Patient Survey. To allow interpretable, annual comparisons the results presented are, therefore, based on annual denominators of all patients (for THIN) and all respondents (Patient Survey). However, between 2004 and 2005, the period when the gap between patient-reported and documented advice appears in Figure 1, there was little change in smoking prevalence in England[12], and thus changes in prevalence are unlikely to explain the divergent data.

This study is limited by a lack of data on patients' recall of smoking cessation advice prior to 2004 (the first Patient Survey, in 2003, asked whether respondents had *tried *to get help to quit smoking from local health care services rather than whether they had received cessation support at their GP surgery). One explanation for the findings reported here is that patients' propensity to recall advice may have changed over time - in the latter years of this study patients may simply have found cessation advice from health care professionals less memorable. However, for diminished recollections of advice to explain findings, patient recall would have to have diminished quite substantially in a very short period, so it seems likely that other reasons account for the difference.

The relatively low Patient Survey response rates raise the possibility of response bias, with smokers or patients recalling advice perhaps being more or less likely to complete the survey; if the latter is the case, the Patient Survey may underestimate the proportion of patients who recall having received cessation advice. However, the response rates in 2004 and 2005 are very similar and the characteristics of respondents completing the survey in these two years are unlikely to have changed substantially. Again, therefore, it seems reasonable that other factors also account for the divergence in recorded and recalled advice rates.

The findings presented here are contrary to those from research studies, which showed more patients recalling receiving advice than had this documented in their medical records[13]. Historically, GPs may not have documented all cessation advice delivered to smokers, though when asked a majority claim to have done this[14]; with the introduction of the QOF, from 2004 onwards GPs may simply be documenting more of the advice that they give[5]. The failure to observe large increases in patients' recall of cessation advice, alongside no increase in rates of prescribing of stop smoking medications[5], tends to support this. The divergence between rates of recording of advice and patient recall seen in Figure 1 is less easy to explain, unless there was an increase in the amount of advice being delivered in such a way that patients did not perceive it as such. GPs have different approaches to advice giving[15], and thus advice documented in patient records could reflect simply the briefest mention of smoking and not be of sufficient duration or intensity to be recalled as 'advice' by smokers[16]. Alternatively, GPs could be recording offers of advice that were not actually made or which were refused; if the latter occurred, patients would not necessarily report receiving advice whereas the offer could legitimately be recorded in their medical records. To our knowledge there is no research which validates smoking cessation Read Codes, so it is not possible to be sure of the degree to which codes used in routine clinical practice represent the nature and extent of the advice delivered to smokers; this is an area for further research.

In conclusion, this study finds substantial increases in the number of patients with a record of cessation advice having been delivered in their primary care medical records, and a smaller increase in the proportion reporting having received advice. However, the discrepancies between these data sources and the inherent difficulties involved in interpreting each mean we cannot be sure that, since the introduction of the QOF, the proportion of smokers being advised to quit by primary care health professionals has actually improved as much as the improved documentation rates suggest. Our findings call into doubt the effectiveness of the financial incentives introduced to encourage GPs to deliver cessation advice to smokers and add to the limited body of evidence evaluating the effect of pay for performance schemes on clinical care and patient outcomes[17].

## Competing interests

In the last 5 years, Tim Coleman has been paid for consultancy work by Johnson and Johnson and Pierre Fabre Laboratories (manufacturers of nicotine replacement therapy). However, this manuscript has not been discussed with any third parties.

## Authors' contributions

LS conceived the study, performed the statistical analyses and wrote the first draft of the manuscript. All authors contributed to its critical revision and approved the final version.

## Pre-publication history

The pre-publication history for this paper can be accessed here:

http://www.biomedcentral.com/1471-2458/11/291/prepub
